# Relationship between the lateral acromion angle and postoperative persistent pain of distal clavicle fracture treated with clavicle hook plate

**DOI:** 10.1186/s13018-020-01737-z

**Published:** 2020-06-11

**Authors:** Kailun Wu, Xinlin Su, Stephen J. L. Roche, Michael F. G. Held, Huilin Yang, Robert N. Dunn, Jiong Jiong Guo

**Affiliations:** 1 Department of Orthopedics, The First Affiliated Hospital of Soochow University, 188 Shizi St, Suzhou, 215006 China; 2 Department of Orthopedics, Suzhou Dushuhu Public Hospital, The First Affiliated Hospital of Soochow University Dushuhu Branch, Suzhou, China; 3 Orthopaedic Research Unit, Department of Orthopaedic Surgery, Groote Schuur Hospital and Red Cross Children’s Hospital, University of Cape Town, Cape Town, South Africa

**Keywords:** Distal clavicle fractures, Clavicle hook plate, Lateral acromion angle, Distal clavicle–acromion coronal angle, Subacromial impingement, Rotator cuff lesion

## Abstract

**Background:**

The clavicular hook plate is an accepted surgical procedure for distal clavicle fractures. The relationship of the characteristics of the hook plate, acromioclavicular joint and acromion morphology, and clinical outcome has remained poorly understood. We reviewed the clinical records of patients who had distal clavicle fractures with different lateral acromion angles treated using a clavicle hook plate and evaluated their clinical outcomes with respect to shoulder pain and acromial morphology.

**Methods:**

We retrospectively reviewed 102 patients with distal clavicle fractures treated with hook plates at our institution from 2010 to 2017. They were divided into four groups according to lateral acromion angle on shoulder AP view X-rays. The angle was defined as the incline angle between the superior surface of distal clavicle and the inferior facet of acromion on coronal plane. We reviewed their clinical features, including Neer’s impingement sign, MRI findings, and outcomes using Japanese Orthopaedic Association Scores. The mean follow-up was 25.5 months (range, 24 to 28 months).

**Results:**

All patients in group D (large lateral acromion angle (α) > 40°, acromion coronal angle (β) < 60°) complained of postoperative symptoms. Compared to those with common lateral acromion angle, the incidence of postoperative impingement in group D was undoubtedly much higher (100%). Japanese Orthopaedic Association (JOA) scores in group D were worse at 3 months post-surgery, 3 months post plate removal, and at the last follow-up despite a slightly earlier removal in this group.

**Conclusion:**

Lateral acromion angle appears to be an important factor in the development of postoperative pain and worse outcomes (JOA scores) in patients treated with the hook plate. The incidence of subacromial impingement and rotator cuff lesion (RCL) increased with the α angle. Early limited mobility and removal of the implant may improve the prognosis and resolve the postoperative shoulder pain.

**Study design:**

Retrospective review, level of evidence IV.

## Introduction

Distal clavicle fractures are usually caused by indirect violence and account for approximately 21% of all clavicle fractures [1]. They are divided into three types according to the relationship of the fracture line to the coracoclavicular ligaments and acromioclavicular joint by Neer [2]. These fractures have been documented to have a significant non-union rate (as high as 22–31%) when treated conservatively, particularly Neer type II [3]. Surgery has been recommended and been shown to reduce this non-union rate and improve clinical outcomes [3].

There is an array of surgical options in the literature including K-wire transfixation [4], tension band wires [5], coracoclavicular screw fixation [6], ligament repair or reconstruction [7], and clavicular hook plate. The clavicular hook plate is a popular surgical treatment, which can provide high stability for the acromioclavicular joint. Clinical research has demonstrated stable fixation, good union rates, and few complications [8]. However, there are well-documented complications such as acromial osteolysis, subacromial shoulder impingement, rotator cuff tears, and subacromial pain [9, 10].

Clinical studies have reported this postoperative pain and suggested it is related to subacromial shoulder impingement and rotator cuff lesions (RCL) due to the position/type of implant [10, 11]. They have recommended that it was necessary to remove the hook plate as soon as bony union was achieved [10, 11].

We believe that postoperative pain may be more closely related to the morphology and complex structure of the plate, distal clavicle, and acromion, and not just the presence of the hook plate itself in the subacromial plate. Other authors have suggested that the morphology of the acromioclavicular joint and impingement is based on the sagittal diversity of acromioclavicular joint seen in the general population [12–14].

The present study is to explore the relationship of the angle of the acromion to the clavicle and clinical outcomes specifically postoperative pain and impingement. We defined a large lateral acromion angle as a distal clavicle–acromion coronal angle > 40° and an acromion coronal angle < 60°. In addition, we provided a new method for measuring the distal clavicle–acromion coronal angle, which could make it more efficient in the application of the clavicular hook plate.

## Materials and methods

### Patient characteristics

From January 2010 to August 2017, 129 patients who sustained displaced distal clavicle fractures and presented to our trauma center (Department of Orthopedics, The First Affiliated Hospital of Soochow University) were identified from our database. All preoperative X-ray showed the obvious fracture displacement. With the failure of manual reposition, internal fixation must be performed on a reduced and aligned fracture. All patients underwent surgery using the clavicular hook plate for fixation. This study was approved by the Ethics Committee of our hospitals, and all patients provided written informed consent.

Fifteen patients were excluded from the study due to one or more of the following: (1) bilateral clavicular fractures, (2) fractures that underwent prior surgery, (3) severe additional associated shoulder injuries, (4) abnormal shoulder function prior to injury, (5) type III fractures (classified according to the Neer classification: Type I occurs lateral to the coracoclavicular ligaments, type II is characterized by a medial fracture with the coracoclavicular ligaments ruptured, type III is an intraarticular fracture of the acromioclavicular joint). Twelve were lost to follow-up within the 2 years.

**Fig. 1:**
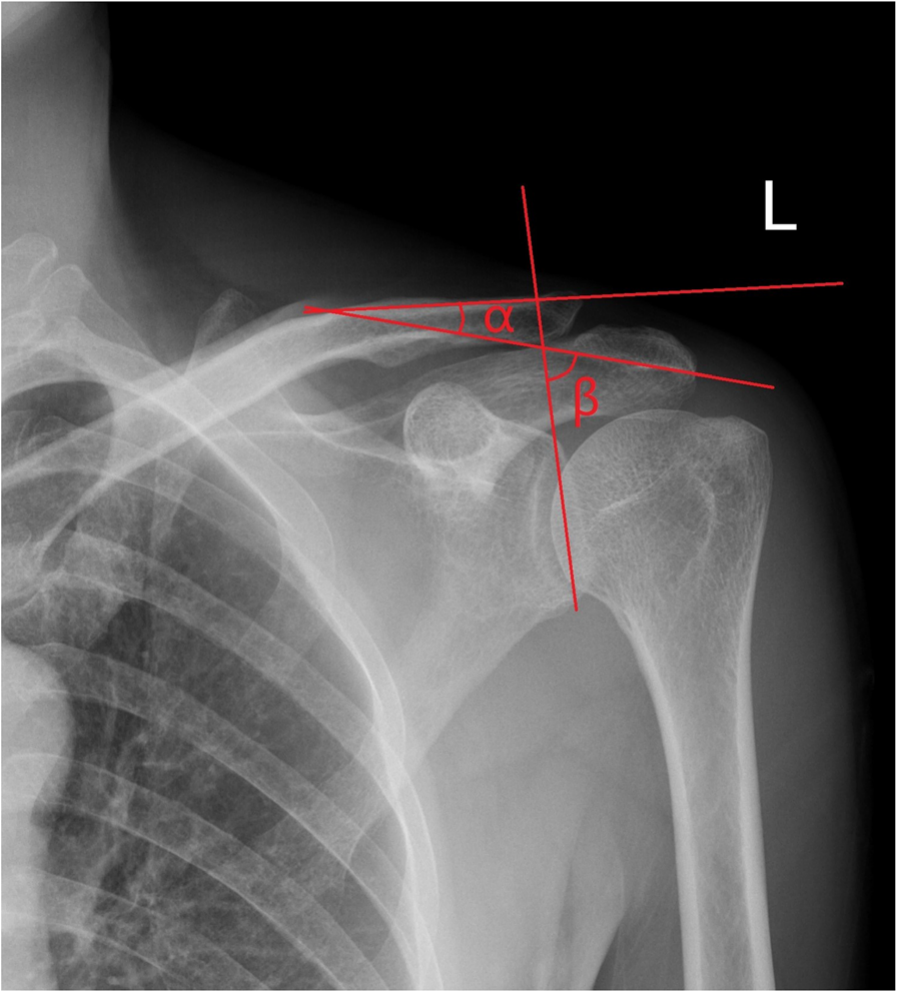
Calculate the distal clavicle–acromion coronal angle (α) and acromion coronal angle (β) on a standardized true anteroposterior radiograph

We defined the distal clavicle–acromion coronal angle (α) as the incline angle between the upper surface of distal clavicle and the inferior facet of acromion on coronal plane, which was consistent with the practical situation of implanting the clavicular hook plate. The acromion coronal angle (β) was then measured as the angle between a line drawn along the inferior facet of acromion and the line from the superior and inferior margin of the glenoid cavity on coronal plane (Fig. 1). Of note, when the acromial under-surface was uneven to the extent that a parallel line cannot be determined, the under-surface line was drawn through the most medial and lateral points of the inferior acromion. Similarly, for the large deformation shaft of distal clavicle, the upper-surface line was drawn through the points marking the most lateral aspect and the corner of the superior clavicle. All radiographs were evaluated at the end of the study by one observer (XS), who was blinded to the outcome.

**Fig. 2:**
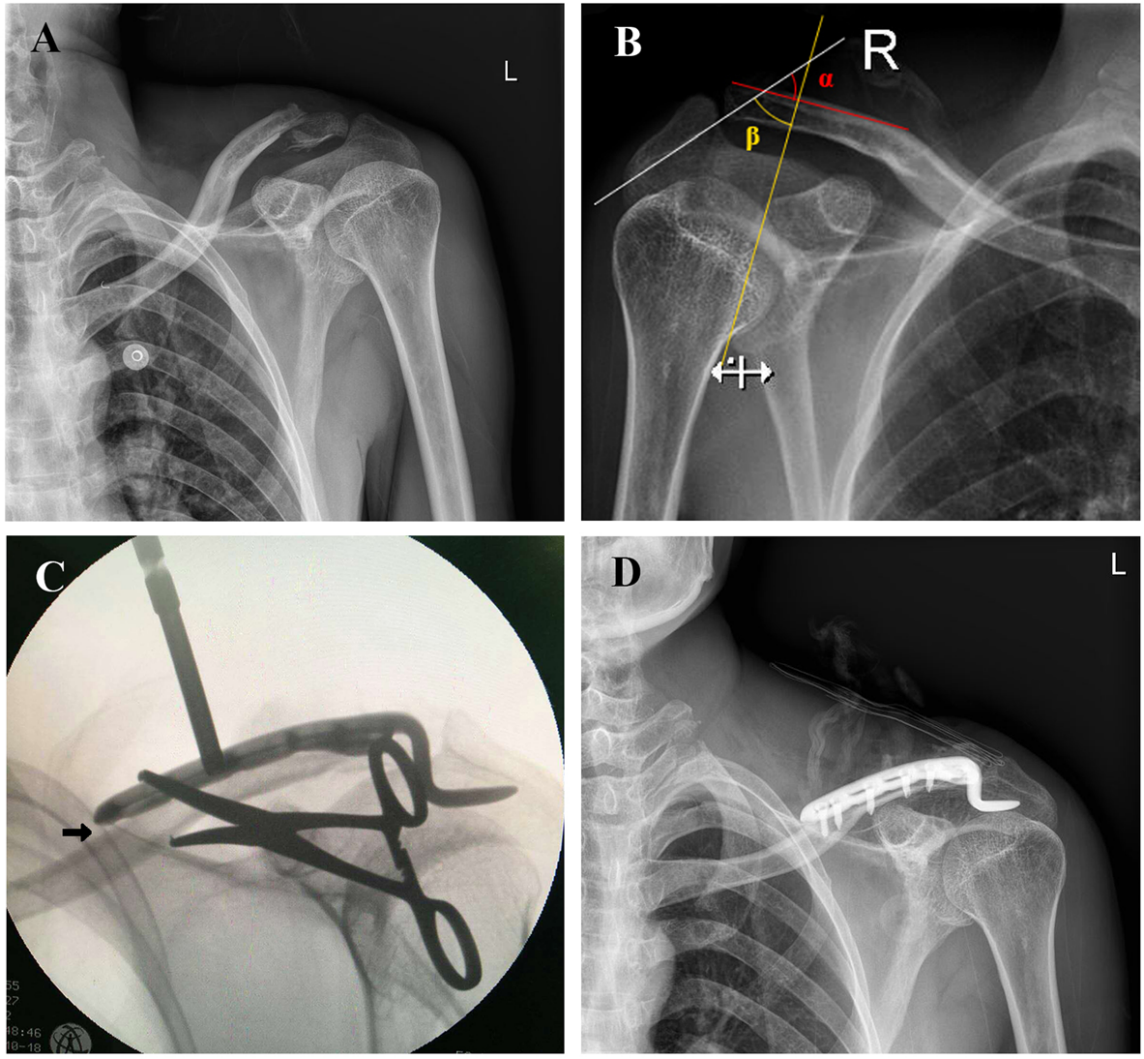
Case 1: A 30-year-old man with left distal clavicle fracture treated with clavicular hook plate. **a** Preoperative radiography indicated Neer type I fracture of the distal clavicle. **b** distal clavicle–acromion coronal angle (α) and acromion coronal angle (β) on X-ray films of uninjured side. **c** Intraoperative fluoroscopy showed the endpiece of plate uplifted (black arrow). **d** Postoperative X-ray demonstrated accepted fracture reduction

There were nine cases with large lateral acromion angle as group D. The common characteristics of these patients included a distal clavicle–acromion coronal angle (α) > 40°, acromion coronal angle (β) < 60°, and the inferior facet of acromion being particularly wide referring to the uninjured side (Fig. 2a, b). The other 93 patients who had distal clavicle fractures with common lateral acromion angle (α < 40° and β > 60°) were compared. In order to better describe it, they were divided into three groups based on angular variation (group A 0° < α < 20° and 70° < β < 90°; group B 20° < α < 30° and 60° < β < 80°; group C 30° < α < 40° and 60° < β < 70°). Classification and measurement of the acromioclavicular joint index were made at standardized true anteroposterior radiographs using X-rays with a resolution of 0.1 mm. The above mentioned morphological parameters were measured using Digimizer Image Analysis Software.

### Surgical procedures

All surgeries were performed by trauma trainees at our orthopedic trauma center (The First Affiliated Hospital of Soochow University) within 1 week (mean time, 3.2 days) after injury. Surgery was performed under general anesthesia and in the beach-chair position. A linear incision was made on the distal clavicle. After reduction and confirming of the subacromial space, the hook plate was placed beneath the acromion posterior to the acromioclavicular joint. Two different depths of hook (15 mm and 18 mm) were available. However, during surgery, a hook type of 15 mm has difficulty in accommodating the thicknesses of the acromion with a large lateral acromion angle. Unexpectedly, the fracture could not be reduced after positioning the clavicular hook plate in group D, which manifested as the endpiece of the plate uplifted or a fracture shift (Fig. 2c). Taking the contours of the bone into consideration, we tried a number of implants with different angles (90°, 95°, 100°, 105°, and 110°). No matter how we configured the plate to the shape of the clavicle, it turned out that the resistance of reduction increased with the bending angle reducing.

### Postoperative management

The shoulder was kept in a shoulder immobilizer for 1 month. Passive pendulum exercises were encouraged 3 days postoperatively with the aid of the uninjured arm. Ice compression was applied after exercise. Active exercises were started at 3 weeks post-surgery. Patients were required to take active exercise after 3 weeks. Active motion of over 90° was permitted at 6 weeks post-surgery. Patients were followed up 3 monthly with X-rays and were scored using Japanese Orthopaedic Association (JOA). After the fractures healed, the patients were allowed to have removal of the hook plate.

### Clinical assessment

Subacromial impingement was diagnosed according to Neer’s impingement sign. The Japanese Orthopaedic Association (JOA) scoring system was used for functional assessment at 3 months after internal fixation and 3 months after removal of fixation [15]. The JOA score is based on 100 points including pain assessment (30 points), shoulder function (20 points), range of movement (ROM) (30 points), radiographic evaluation (5 points), and joint stability (15 points). In accordance with the JOA shoulder assessment by Tomoya et al. [16], we defined functional recovery as a score more than 80% in each JOA shoulder assessment component. Magnetic resonance imaging (MRI) was performed if constant pain remained for 3 months after bony union and plate removal. The rotator cuff lesion is based on critical identification of the MRI for the changes seen in rotator cuff tissue signal and morphologic appearance. Rotator cuff tear refers to a supraspinatus tendon with both abnormal signal and morphologic appearance with or without a definite area of discontinuity within the tendon.

### Statistical analysis

All data were analyzed using the SPSS statistical package (version 20.0; SPSS Inc.) and represented as mean and standard deviation (SD) for continuous response variables, or numbers and percentages for discrete variables. The independent samples *t* test was used for analysis of continuous variables. The level of significance was set at *P* < 0.05.

## Results

In group D, the mean of α angle was 44.6°, while that of β angle was 53.4°. Hooks with a 15-mm depth were used in four cases, and in three cases, a big angle hook with exceeding 100° was used. JOA Scores at 3 months postoperatively (mean 65.8) was lower than that at 3 months (mean 75.7) and 1 year (mean 79.1) after removal. However, one patient (case 1) was an exception before and after the removal with no difference. Radiography at the immediate postoperative period showed good fracture reduction. According to clinical and radiographic results, all fractures achieved bony union within 3 months after surgery. The clinical and demographic data were shown in Tables 1 and 2.

**Table 1:** Summary of our cases of distal clavicle fractures with large lateral acromion angle

Case no.	Age, year/sex	Side	α	β	Specification (depth and angle of hook, mm)	Postoperative recovery				Total follow-up time (month)	Treatment and outcome
						JOA Scores (3 months postoperatively)	Time of hardware retention (month)	JOA Scores (3 months after removal)	JOA Scores (1 year after removal)		
1	30/M	Left	49.1	40.6	15, 90°	62	5	60	65	24	Untreated, lost to follow-up
2	52/F	Right	44.7	52.4	15, 95°	65	6	75	76	28	RCR, remission
3	27/F	Right	41.5	56.8	18, 110°	55	7	75	77	24	RCR, remission
4	44/M	Right	47.8	54.8	15, 90°	50	8	62	70	27	RCR, remission
5	46/F	Right	45.1	44.5	18, 100°	87	7	95	96	25	remission
6	39/F	Left	44.1	59.7	18, 90°	65	5	70	75	24	RCR, remission
7	38/M	Left	42.2	53.5	15, 90°	83	7	96	94	24	remission
8	55/F	Right	40.4	58.9	18, 100°	70	6	82	87	26	Untreated, partial remission
9	56/M	Right	46.2	59.6	18, 90°	54	8	66	72	24	RCR, remission

*M* male, *F* female, *JOA* Japanese Orthopaedic Association, *RCR* rotator cuff repair

**Table 2:** General clinical data of cases of distal clavicle fractures with common and large lateral acromion angle

		*N*	Age, year	Sex, M/F	α	β	Neer type (I/II)	Total follow-up time (month)
Cases with common lateral acromion angle	Group A	56	39.4 ± 9.6	25/31	16.65 ± 1.8	83.34 ± 3.8	11/45	25.6 ± 1.2
	Group B	21	42.6 ± 10.1	12/9	23.86 ± 2.1	75.15 ± 3.2	4/17	25.8 ± 1.1
	Group C	16	44.3 ± 9.3	9/7	34.12 ± 2.5	64.62 ± 4.3	2/14	26.3 ± 1.2

All variables are presented as mean ± SD except sex, Neer type

*M* male, *F* female, *N* number of patients

**Fig. 3:**
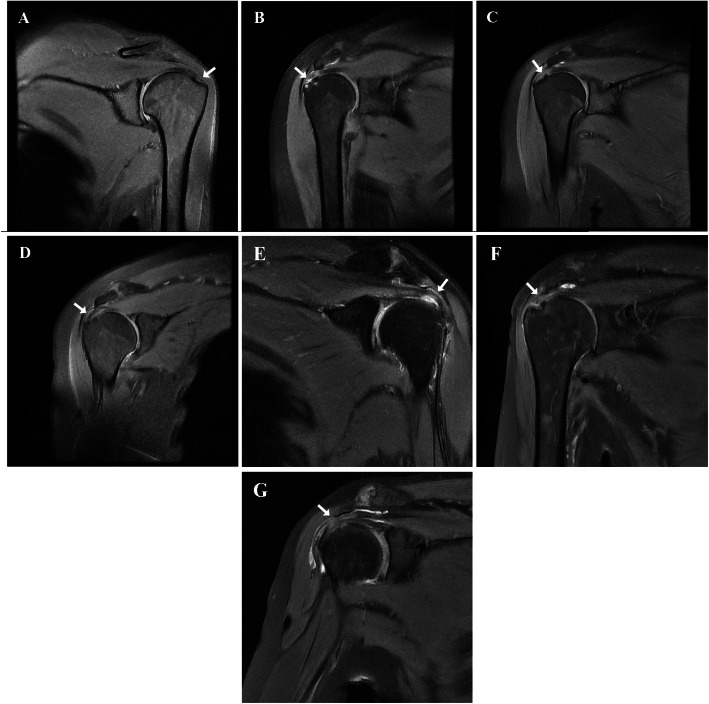
**a**–**g** Cases 1 ~ 4, 6, and 8 ~ 9: Preoperative sagittal T2-weighted magnetic resonance (MR) imaging reveals the interruption of supraspinatus tendon continuity (white arrows)

Postoperatively, all patients in group D complained of implant-related symptoms including pain, scraping feeling, and limited motion of the operated shoulder. The clinical subacromial impingement (positive Neer’s sign) was observed in all. Nevertheless, cases 5 and 7 had relief gradually within approximately 2 months after implantation. The symptom of case 8 was relieved significantly after withdrawal of the plate. In contrast, unbearable pain was noted for daily life and work in the remaining 6 patients with the range of JOA from 50 to 65 points. Their symptoms did not subside (strength and ROM of shoulder was slightly improved, but with no significant differences in pain), even if undergoing early removal of the plate like case 1. A RCL was further confirmed eventually with the aid of MRI examination in these cases (Fig. 3), five of whom received rotator cuff repair and achieved remission. At final follow-up, case 1 refused a second surgery and was lost to follow-up.

**Fig. 4:**
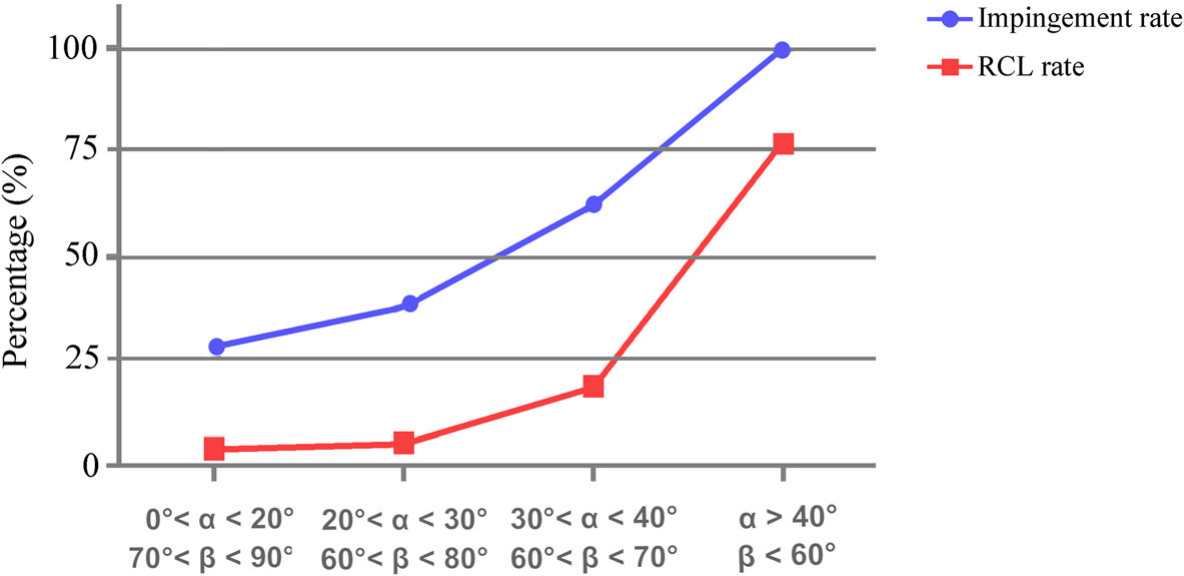
Graphic presentation of impingement and RCL rates correlated with α and β angles

Of the 93 patients with common lateral acromion angle, eight (six in group A and two in group B) refused to remove the internal fixation owing to advanced age or family factors so that their conditions of rotator cuff cannot be detected. The mean time of hardware retention of the remaining patients was 7.9 months in group A, 7.7 months in group B, and 7.3 months in group C. Of particular note was the exponential growth of RCL rate as the α angle increased. It was not hard to see that a large lateral angle (α = 40°; β = 60°) was a dividing line from Fig. 4, which was consistent with the higher frequency of impingement. Moreover, from the perspective of JOA scores, postoperative pain and function were of significant difference among them. With the time extending, the prognosis for the patients in groups A and B has got a lot better, compared with group D. The clinical data of patients with common lateral acromion angle was recorded in Table 3.

**Table 3:** JOA Scores and postoperative clinical data of cases of distal clavicle fractures with common and large lateral acromion angle

		*N*	Postoperative recovery				Impingement rate and RCL rate
			JOA Scores (3 months postoperatively)	Time of hardware retention (month)	JOA Scores (3 months after removal)	JOA Scores (1 year after removal)	
Cases with common lateral acromion angle	Group A	50	82.5 ± 7.8	7.9 ± 1.5	86.9 ± 8.5	90.5 ± 6.9	16/56 (28.6%) and 2/50 (4.0%)
	Group B	19	80.4 ± 8.5	7.7 ± 1.5	88.6 ± 8.2	90.8 ± 7.1	8/21 (38.1%) and 1/19 (5.3%)
	Group C	16	77.6 ± 9.1	7.3 ± 1.5	83.1 ± 8.0	85.3 ± 7.2	10/16 (62.5%) and 3/16 (18.8%)

All variables are presented as mean ± SD except Impingement rate & RCL rate

*N* number of patients, *JOA* Japanese Orthopaedic Association, *RCL* rotator cuff lesion

Interestingly, when we used “6 months” as a dividing line of the removal time, the results suggested that early removal of internal fixation could achieve a higher JOA Score 1 year after removal and a lower rate of impingement. It was worth mentioning that above difference turned out to be not significant in terms of JOA Scores at 3 months after surgeries. Furthermore, we also observed no significance regarding RCL rate (Table 4).

**Table 4:** JOA Scores and postoperative clinical data of cases with different time of hardware retention

Time of hardware retention (month)	*N*	Postoperative recovery			Impingement rate	RCL rate
		JOA Scores (3 months postoperatively)	JOA Scores (3 months after removal)	JOA Scores (1 year after removal)		
≤ 6	28	79.3 ± 6.7	86.5 ± 7.5	90.9 ± 7.4	9/28 (32.1%)	4/28 (14.3%)
> 6	66	79.8 ± 7.7	85.1 ± 5.6	87.6 ± 5.9	30/66 (45.5%)	9/66 (13.6%)
*P*	–	0.739	0.212	0.049*	0.009*	0.870

All variables are presented as mean ± SD except Impingement rate and RCL rate

*N* number of patients, *JOA* Japanese Orthopaedic Association, *RCL* rotator cuff lesion

*P < 0.05

## Discussion

Several recent studies have suggested that the use of the clavicular hook plate is the best method for distal clavicle fracture, especially for Neer type II fracture, with respect to the achievement of rigid fixation and a high rate of fracture union [3, 9]. Karduna et al. proved that the hook plate can provide stronger anti-deformation capacity than conventional fixation such as tension band wire [17]. However, other researchers believe that the hook plate can exert adverse effects on subacromial tissues, including subacromial impingement, acromial osteolysis, and rotator cuff tear. A cadaver study showed that the placement of the implant should be positioned according to the different types of acromion, because the positioning may cause subacromial impingement [13]. Another cadaver study also indicated that the designs of the hook plate have still not addressed the difference of acromioclavicular joint morphologies [18]. All the above suggested that appropriate selection of the clavicle hook plate correlated with outcomes. Only by combining the three factors of characteristics of hook plate (plate length, hook depth, and hook angle), fracture pattern, and acromioclavicular joint morphology can these problems be averted.

The literature has still lacked a clear understanding of the relationship between characteristics of hook plate and acromioclavicular joint morphology. To date, almost all studies focused on the importance of acromion sagittal angle and neglected distal clavicle–acromion coronal angle [12, 19, 20]. Furthermore, conventional distal clavicle–acromion angle (measured from the central axis of the distal clavicle and acromion) was insufficient for practical requirements according to our experience. Therefore, we undertook this review of our patients to determine whether the different lateral acromion angle correlated with the severity of postoperative pain in treating distal clavicle fractures with a clavicular hook plate.

### Type selection of clavicular hook plate

Currently, clavicular hook plates still do not match the anatomy of the distal clavicle and acromion perfectly [21]. For remedying the situation, a number of alternative characteristics of hook plate during the procedure, such as different plate length, hook depth, and hook angle, have been recommended. However, for some rare acromial morphology like large lateral acromion angle, these characteristics cannot meet our demands.

**Fig. 5:**
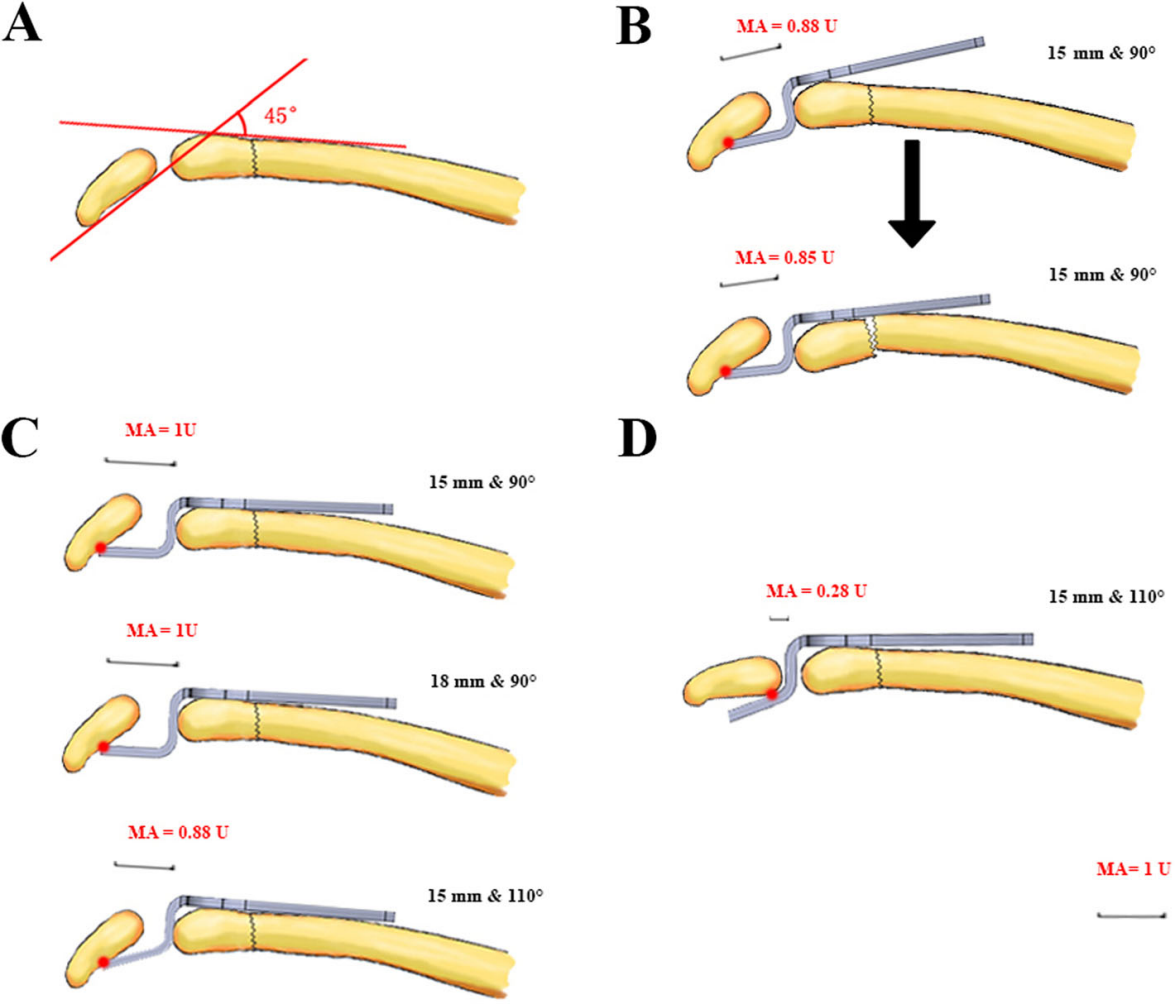
**a**–**c** The situation of dynamically simulating the changes of contact position between the hook plate and large lateral acromion angle. **a** Take 45° α angle for example and length of hook as one unit of moment arm (MA). **b** The change of contact position (red dot) and fracture shift when pressing hook plate (15 mm and 90°). **c** The change of MA when using different hook specifications. **d** The contact position (red dot) and MA between the 110°-hook plate and normal lateral acromion angle

In our study, only a plate equipped with a depth of 18 mm and big angle can barely accommodate the acromion with a large lateral acromion angle. Interestingly, as the angle of implant decreased, the resistance of reduction increased, which cannot be weakened by reshaping the plate. In our experience, this phenomenon was reflected obviously when α > 30° and β < 70°. Lee and Shih et al. investigated the mechanics of the plate length and hook depth using finite element analysis (FEA) method and found that the stress on the acromion and clavicle was smaller when using a hook plate with greater length and depth [22, 23]. Hung et al. also used FEA to investigate the impacts of different hook angles. They found that a larger hook angle of implant exerted a larger load on the acromion because the larger hook angle made the contact position between the hook plate and acromion more proximal [24]. This theory seemed to accord with the phenomenon we encountered. On the contrary, the contact position between the hook plate and acromion was in fact away from the proximal when using a small angle hook in large lateral acromion angle (Fig. 5). Therefore, the hook plate was forced to be attached to the distal and proximal part of the clavicle simultaneously, leading to excessive stress. As to the depth of hook, we found that different depths would change the counterforce of acromion by the method of shoving acromion rather than the moment arm. The hook of 18 mm depth may withstand smaller stress.

Paradoxically, a greater depth of hook plate may induce mechanical attrition of the supraspinatus aponeurosis more easily. Previous study has shown that the proximal clavicular hook was the main part compressing the supraspinatus tendon [11]. When using a small angle, the corner of the proximal hook was fallen lower, resulting in a larger contact area and force on the supraspinatus. Furthermore, as the distal clavicle–acromion coronal angle increased, the risk of proximal hook slippage may increase [12]. Improper selection and poor understanding of hook specification partially explain the reason for postoperative persistent pain. Therefore, at least in theory, we suggest that other schemes have to be taken into account when the α angle exceeds 20°. Whereas, from the observation in clinical practice, the α angle exceeding 30° may be a clear indication.

### Lateral acromion angle and impingement

In our research, all patients in group D complained of a variety of pain related to impingement, especially when their arms were lifted over their head. In addition, seven patients who had shoulder impingement symptoms scored lower JOA scores and had poor satisfaction. At the end of this study, the impingements had developed into rotator cuff tear in these seven cases for an overall incidence of 77.8%! In our cases with common lateral acromion angle, we have not found such a high rate of RCL, though the incidence had a tendency to increase with an increase in α angle.

Other investigators have also reported subacromial impingement and rotator cuff tear associated with implantation of a clavicular hook plate [9–11, 13, 25–30] (Table 5). Of particular concerns were two articles. One study reported that incidence of subacromial shoulder impingement and RCL was calculated by dynamic sonographic evaluation, reaching as high as 37.5% and 15% [10]. Another one investigated 12 patients treated with hook plate by arthroscopic evaluation, and 91.7% of them developed signs of impingement [28]. Compared with the above, there was a far higher incidence of pain and rotator cuff tear in group D. We considered that the stress on supraspinatus tendon increased on account of the large lateral acromion angle. Subacromial space could further narrow the distance between the supraspinatus tendon and the base of subacromial hook due to the large lateral acromion angle. Meanwhile, the supraspinatus tendon and bursa could be susceptible to irritating friction when the arm was elevated. Almost all studies have neglected the effect of distal clavicle–acromion coronal angle in the treatment of distal clavicular fracture, which was a significant factor impacting the outcome of the hook plate in our study. Our research also revealed that the rate of impingement and RCL had a tendency to grow exponentially with the increasing of the α angle (Fig. 4). However, this trend cannot be represented very precisely due to uneven distribution of the angular variation.

**Table 5:** Summary of reported studies related subacromial impingement treating with clavicular hook plate

Author, reference	Year	Study design	Case no. (M/F)	Duration of follow-up (mean, month)	Impingement rate and RCL rate	Hardware removal (time after fixation operation, *N*)
Kashii et al. [23]	2006	Case series	34 (28/6)	12.4	2/34 (5.9%)	5.3
Muramatsu et al. [24]	2007	Case series	15 (13/2)	15.5	0/15 (0%)	4.5, 12
Meda et al. [25]	2006	Case series	31 (24/7)	40	6/31 (19.4%)	5.56
Renger et al. [8]	2009	Case series	44 (29/15)	27.4	33/44 (75%)	8.4
Lee et al. [26]	2009	Case series	32 (14/18)	26.4	0/32 (0%)	4.8, 32
ElMaraghy et al. [12]	2010	Cadaveric studies	15 (7/8)	NA	9/15 (60%)	NA
Hsu et al. [27]	2010	Case series	35 (23/12)	6	9/35 (25.7%) and 0/35 (0%)	12, 35
Leu et al. [28]	2012	Case series	25 (13/12)	14.5	9/25 (36%)	5.8, 25
Lin et al. [9]	2014	Case series	40 (30/10)	13.6	15/40 (37.5%) and 6/40 (15%)	5.78, 40
Gu et al. [10]	2014	Case series	12 (7/5)	NA	11/12 (91.7%) and 1/12 (8.3%)	NA, 12
Our cases	–	Case series	9 (4/5)	23	9/9 (100%) and 7/9 (77.8%)	6.7, 9

*M* male, *F* female, *N* number of patients, *NA* not applicable, *RCL* rotator cuff lesion

To the best of our knowledge, the only solution to postoperative persistent pain was removal of the implant as soon as a bony union has occurred. Leu et al. demonstrated that the impingement problems can disappear within 8 weeks after removal [30]. Of our patients, the mean time of removal ranged from 6.6 to 7.8 months, which was longer than the other studies. A shorter interval between union and plate removal might provide a key factor for preventing further development of impingement. As evidenced by our supplementary research, the rate of impingement was considerably lower for those undergoing a removal surgery within half year after operation. But the rate of RCL cannot be supported by this comparison. In addition, early excessive mobility of the acromioclavicular joint may be another reason for developing a RCL in patients with a large lateral acromion angle. According to a study by Kashii et al. [25], patients should avoid forward flexion or adduction greater than 90° and internal rotation of the shoulder behind the back until the hook plate is removed.

A limitation of this study is the small number of patients with a large lateral acromion angle. Similarly, while one may assume the impingement from the plate cause the rotator cuff tear, this study, as it is designed, cannot establish causation. In order to establish causation, the patients each would have had to have an ultrasound or MRI before fracture fixation or the injury. But it is unlikely to perfect interrelated examinations before the injury. Also, angles measured on trauma X-rays may not always be that reliable. The precise analysis of angles could be established by comprehensive consideration of the CT appearance. Lastly, a separate analysis of the hook plate was incomplete. Instead, a comprehensive assessment was available only by comparing a variety of procedures.

## Conclusions

A total 102 distal clavicle fractures were treated with clavicle hook plate. Persistent pain caused by repeated impingement or rotator cuff tear occurred. Firstly, although the distribution of the angular variation is uneven, we have demonstrated that the distal clavicle–acromion coronal angle is an important factor for postoperative efficacy of the hook plate. Secondly, the selection of characteristics of hook plate should make the contact position between the hook and acromion more proximal. Thirdly, early limited mobility and removal of the implant may improve the prognosis and reduce the rate of impingement. In our opinion, for patients with a large lateral acromion angle, other surgical fixation should be considered as the hook plate has a high complication rate despite achieving union of the fracture.

## Data Availability

The datasets used and analyzed during the current study are available from the corresponding author on reasonable request.

## Abbreviations

JOA: Japanese Orthopaedic Association ROM Range of movement MRI Magnetic resonance imaging RCL Rotator cuff lesion FEA Finite element analysis MA Moment arm
